# Circular RNA Circ0021205 Promotes Cholangiocarcinoma Progression Through MiR-204-5p/RAB22A Axis

**DOI:** 10.3389/fcell.2021.653207

**Published:** 2021-05-03

**Authors:** Jianfei Tu, Weiqian Chen, Liyun Zheng, Shiji Fang, Dengke Zhang, Chunli Kong, Yang Yang, Rongfang Qiu, Zhongwei Zhao, Chenying Lu, Xiaojie Lu, Jiansong Ji

**Affiliations:** ^1^Key Laboratory of Imaging Diagnosis and Minimally Invasive Intervention Research, Lishui Hospital of Zhejiang University/Fifth Affiliated Hospital of Wenzhou Medical University, Lishui, China; ^2^Clinical College of The Affiliated Central Hospital, Lishui University, Lishui, China; ^3^Affiliated Hospital of Youjiang Medical University for Nationalities, Baise, China; ^4^Department of General Surgery, The First Affiliated Hospital of Nanjing Medical University, Nanjing, China

**Keywords:** cholangiocarcinoma, circular RNAs, circ_0021205, miR-204-5p, RAB22A

## Abstract

Cholangiocarcinomas (CCA) are biliary tract tumors that are often challenging to diagnosis and treatment. Accumulated evidence reveals that circular RNAs (circRNAs) are involved in multiple cancer progression. However, the function of circRNAs in cholangiocarcinoma remains largely unclear. In this study, we found that circ_0021205 expression was up-regulated in CCA and positively correlated with tumor size and TNM stage. To further explore the role of circ_0021205 in CCA, cell functional assays were performed. The results showed that circ_0021205 promoted the proliferation, migration, and invasion of CCA cells. *In vivo* experiments showed that circ_0021205 inhibition reduced tumorigenesis in mice. In addition, mechanisms investigation demonstrated that circ_0021205 exerts its oncogenic function by sponging miR-204-5p to regulate the expression of RAB22A. Overall, this study revealed that circ_0021205 might serve as a potential diagnostic biomarker or therapeutic target for CCA.

## Introduction

Cholangiocarcinoma (CCA) is a highly malignant tumor found in the epithelial cells lining the bile duct, which is categorized according to anatomical location as intrahepatic, perihilar, and distal cholangiocarcinoma (Cunningham et al., 2007; Rizvi et al., 2018). In the past 30–40 years, the global incidence of cholangiocarcinoma has risen to 18% of all liver cancers (Erichsen et al., 2011). At present, the effective treatments of cholangiocarcinoma are surgical resection, liver transplantation, and drug therapy (Kinoshita et al., 2008; Wechagama et al., 2012). However, the therapeutic effects of radiotherapy and chemotherapy are relatively poor. The only effective treatment method is early surgical resection of cholangiocarcinoma (Fidelman et al., 2011; Patop et al., 2019). Therefore, it is urgently needed to further explore the pathogenesis of cholangiocarcinoma to develop new diagnostic and therapeutic methods for CCA.

Circular RNAs (circRNAs) are produced by reverse splicing, which is characterized by covalently closed continuous loops and canonical splicing sites (Jens et al., 2013; Li et al., 2018). Due to the lack of 5′ cap structure and 3′ poly(A) tails, circRNAs have a long half-life, which enables them to resist the conventional linear decay mechanism of RNA (Jeck and Sharpless, 2014; Chen et al., 2017). Previous studies have shown that some circRNAs can bind to specific miRNAs as miRNA sponges (Hansen et al., 2013). In addition, recent studies have shown that circRNAs play a role in multiple cancer progression (Legnini et al., 2017; Yang et al., 2017). Because of the long half-life and resistance to common degradation pathways, circRNAs may serve as the potential biomarkers for cancer (Kulcheski et al., 2016). However, the reports about the mechanism and function of circRNAs in cholangiocarcinoma are limited.

MicroRNAs (miRNAs) are non-coding RNAs which play roles in the negative regulation of gene expression at the post-transcriptional level (Lee and Dutta, 2009; Lu and Rothenberg, 2018). In the past decade, it has been found that miRNAs are closely related to the development of cancer and can play the role of tumor suppressor or promoter (Rupaimoole and Slack, 2017). MiR-204-5p was identified as a tumor-suppressive miRNA, plays a role in cancer development and progression (Zhang et al., 2014). In addition, the deregulation of miRNAs is associated with the progression of many tumors, including cholangiocarcinoma (Wan et al., 2018). However, The regulatory mechanism of circRNA-miRNA network on cholangiocarcinoma is rarely reported.

## Materials and Methods

### Tissues and Cell Lines

Cholangiocarcinomas tissues and adjacent tissues were obtained from Lishui Hospital of Zhejiang University. All patients gave written informed consent before this study start. This study was approved by the ethics committee of Lishui Hospital of Zhejiang University. The clinicopathological characteristics of 27 CCA patients were as shown below:

**Table:** 

CCA patients	27
Sex	
Male	13
Female	15
Age	
≤50	17
>50	10
AFP (ng/ml)	
≤20	15
>20	12

All cell lines were purchased from China Center for Type Culture Collection (CCTCC). Normal biliary cell line HIBEC and CCA cell lines (HCCC-9810, Huh-28, and KMBC) were cultured in Roswell Park Memorial Institute (RPMI) 1640 Medium (Gibco, United States), CCA cell lines QBC939, RBE, and HuCCT1 were cultured in Dulbecco’s Modified Eagle’s Medium (DMEM, Gibco). All medium were supplemented with 10% fetal bovine serum (FBS, Gibco), 100 U/mL penicillin and 100 μg/mL streptomycin.

### RNase R Treatment

Total RNA (10 μg) from HuCCT1 and KMBC cells were incubated with 5 U/μg RNase R (Epicenter Technologies, United States) for 15 min at 37 °C. Subsequently, the treated RNA was reverse transcribed and the level of circ_0021205 was examined by quantitative real time PCR (qRT-PCR) assay. Experiments were repeated for three times.

### Cell Transfection

The si-circ_0021205-1 (GTCTAATTCCTGTGGTGAAGA), si-circ_0021205-2 (ATTGTCTAATTCCTGTGGTGA), miR-204-5p mimic (5′-UUCCCUUUGUCAUCCUAUGCCU-3′), and miR-204-5p inhibitor (5′-AGGCAUAGGAUGACAAAGGGAA-3′) were purchased from Thermo Fisher Scientific (United States). The oe-circ_0021205 pasmids were constructed using pLO5-ciR vectors (Geneseed, China). All cell transfection were performed using Lipofectamine 3000 (Thermo Fisher Scientific, United States).

### RNA Extraction and Quantitative Real Time PCR (qRT-PCR)

Total RNA of CCA tissues and cell lines was extracted using TRIzol reagent (Invitrogen, United States), then the concentration and purity of RNA was measured by NanoDrop 2000 (Thermo Fisher Scientific, United States). The qRT-PCR was performed using SYBR Green qPCR Mix (Takara, Japan). For circRNA and mRNA, GAPDH was used as endogenous reference. For miRNA, U6 was used as endogenous reference. All qRT-PCR reactions were run on the ABI 7500 Fast Real-Time PCR System (Applied Biosystems, United States). The relative expression of RNAs was calculated with 2^–ΔΔ*CT*^ algorithm. All primers used in this study were listed in Table 1. Experiments were repeated for three times.

**TABLE 1:** Primers used in qRT-PCR assay.

**Gene**	**Sequences**
circ_0021205	F: 5′-ACATGTTTGCACTTGTCTTTGACT-3′
	R: 5′-TGTCTTCACCACAGGAATTAGACA-3′
miR-204-5p	F: 5′-GGGAAACAGUAGGAUACGGA-3′
	R: 5′-CAGTGCGTGTCGTGGAGT-3′
GAPDH	F: 5′-CGCTCTCTGCTCCTCCTGTTC-3′
	R: 5′-ATCCGTTGACTCCGACCTTCAC-3′
U6	F: 5′-AGCCCGCACTCAGAACATC-3′
	R: 5′-GCCACCAAGACAATCATCC-3′

### Luciferase Reporter Assay

The pGL3 Luciferase Reporter Vectors (Promega, United States) containing circ_0021205-WT, circ_0021205-MuT, *RAB22A*-WT, or *RAB22A*-MuT sequences were co-transfected with miR-204-5p mimics or NC-mimics into HuCCT1 and KMBC cells. After 24 h of incubation, cells were lysed by 1 × PLB, then the luciferase activities were measured using the Dual-Luciferase Reporter Assay System (Promega, United States). Experiments were repeated for three times.

### Cell Counting Kit-8 Proliferation Assay

3 × 10^3^ HuCCT1 and KMBC cells were seeded into 96-well plates and cultured for 2–4 h. Then added 10 μL CCK-8 reagents (YEASEN, China) to each well. After 2 h of incubation, the optional density (OD) at 450 nm was measured by the Multiskan FC with Incubator (Thermo Fisher Scientific, United States). Experiments were repeated for three times.

### Colony Formation Assay

2 × 10^2^ HuCCT1 and KMBC cells in logarithmic growth phase were seeded into 12-well plates, and cultured for 2 weeks. Then cells were fixed using 4% paraformaldehyde for 15 min and stained by crystal violet staining solution for 15 min. Slowly washed the plates with running water and air-dried. Counted the number of colonies in the plates. Experiments were repeated for three times.

### Transwell Migration and Invasion Assay

For migration assay, the transwell chambers (Millipore, United States) were paved without matrigel mix. For invasion assay, the upper transwell chambers were paved with matrigel mix. HuCCT1 and KMBC cells were suspended in medium without FBS and seeded into the upper transwell chambers. And medium supplemented with 10% FBS was infused into the bottom chambers as cell chemo attractant. After 24 h of incubation, the upper chamber was fixed with 4% paraformaldehyde and then stained by crystal violet staining solution for 15 min. The cell numbers were counted in five different fields under the microscope. Experiments were repeated for three times.

### Western Blot and Immunohistochemistry Staining

Cells were lysed using RIPA buffer containing protease inhibitor (Sangon, China). 30 μg total protein extracted from CCA cells was separated by sodium dodecyl sulfate-polyacrylamide gel electrophoresis (SDS-PAGE), and transferred to polyvinylidene fluoride (PVDF) membranes (Millipore, United States). The membranes were blocked by 5% skimmed milk and incubated with the primary antibodies (anti-Ki-67, 1:1000, ab16667, Abcam, United States; anti-PCNA, 1:1000, ab265609, Abcam, United States; anti-GAPDH, 1:1000, ab9485, Abcam, United States; anti-RAB22A, 1:1000 ab137093, Abcam, United States) overnight at 4°C. And incubated with the HRP-conjugated secondary antibodies (1:10000; Abcam, United States) at room temperature for 1 h. Subsequently, the protein bands were visualized using the Clarity Western ECL Substrate (Bio-Rad, United States). The primary antibodies used in this study were as shown below: (anti-Ki-67, 1: 200, ab16667, Abcam, United States; anti-PCNA, 1:250, ab265609, Abcam, United States).

### Xenografts in Mice

All male BALB/c nude mice were purchased from XX. 1 × 10^7^ HuCCT1 cells stably transfected with si-circ_0021205 or si-NC were subcutaneously injected into the mice, respectively. After the injection, we measured the tumor and calculated the tumor volume every 4 days. 20 days after injection, the mice were euthanized, then tumors were excised and measured. We then used the tumor tissues for qOCR, WB, and IHC analysis.

### Statistical Analysis

GraphPad Prism 8.0 (GraphPad Software, San Diego, CA, United States) and SPSS 22.0 (IBM, Chicago, IL, United States) were used in this study. Student’s *t* test and One-way ANOVA followed by Dunnett’s multiple comparisons test were used to analyze the difference between groups. *P* < 0.05 was considered statistically significant. Each experiment was triplicated.

## Results

### Circ_0021205 Is Upregulated in CCA

**FIGURE 1:**
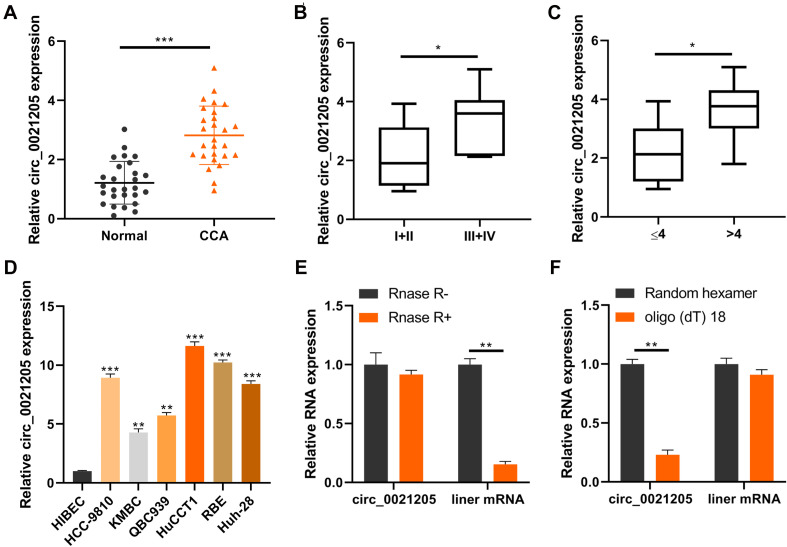
Circ_0021205 is upregulated in CCA. **(A)** QRT-PCR data showed that circ_0021205 was significantly upregulated in CCA tissues than controls. **(B,C)** QRT-PCR data showed that circ_0021205 expression in ≥4 cm size or I + II stage tumor tissues was higher than in small size or III + IV stage tumors. **(D)** The relative expression of circ_0021205 in different cell lines was detected using qRT-PCR. **(E)** QRT-PCR data showed that circ_0021205 expression has not been decreased after RNase R treatment. **(F)** QRT-PCR data showed that circ_0021205 could not be amplified by oligo(dt) 18 primers. All experiments were repeated at least three time. *P < 0.05, **P < 0.01, ***P < 0.001.

To investigate the correlation between circ_0021205 expression and CCA development, we collected 27 pairs of CCA tissues and adjacent tissues, and detected the circ_0021205 expression using qRT-PCR. As shown in Figure 1A, the expression of circ_0021205 in CCA tissues was significantly upregulated compared with that in normal tissues. We also analyzed the circ_0021205 expression in CCA tissues with different size or TNM stage (Figures 1B,C). The results showed that the circ_0021205 expression in larger size or advanced stage tumor tissues was higher. In addition, the expression of circ_0021205 in CCA cell lines was also higher than in normal human intrahepatic biliary epithelial cells (Figure 1D). HuCCT1 and KMBC cell lines were selected for further study for the relative high or low expression level of circ_0021205 among 6 CCA cell lines. To verify the circular feature of circ_0021205, RNase R treatment was performed to examine the stability of circ_0021205 (Figure 1E). Random hexamer and oligo(dt) 18 were used to amplify circ_0021205 and liner mRNA (WEE1) respectively (Figure 1F), the results showed that circ_0021205 is a circular RNA.

### Circ_0021205 Promotes the Proliferation, Migration, and Invasion of CCA Cells

**FIGURE 2:**
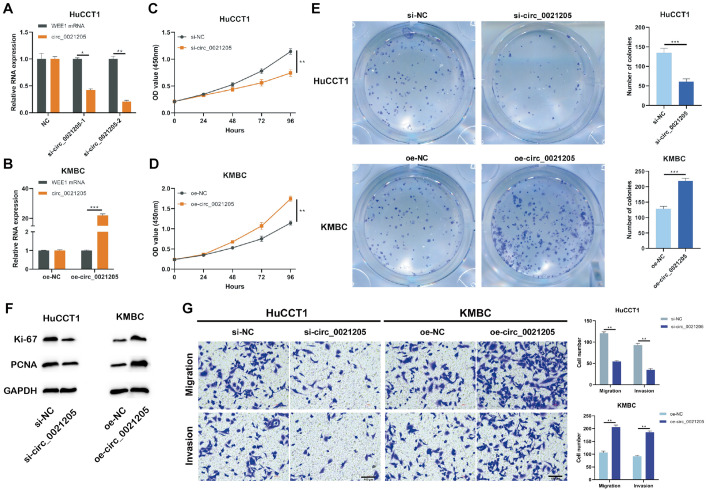
Circ_0021205 promotes the proliferation, migration and invasion of CCA cell. **(A,B)** HuCCT1 cells were transfected with si-NC, si-circ_0021205-1, or si-circ_0021205-2. KMBC cells were transfected with oe-NC or oe-circ_0021205. The transfection efficiencies were analyzed by qRT-PCR. **(C,D)** The proliferation of transfected HuCCT1 and KMBC cells were determined by CCK-8 assay. **(E)** Colony formation assay were performed to detect the proliferation of HuCCT1 and KMBC cells transfected with si-circ_0021205 and oe-circ_0021205, respectively. **(F)** The levels of proliferation makers, Ki-67 and PCNA, were investigated using western blotting. **(G)** Cell migration and invasion were detected using transwell assay. All experiments were repeated at least three time. *P < 0.05, **P < 0.01, ***P < 0.001.

To explore the role of circ_0021205 in CCA, the expression of circ_0021205 was down-regulated in HuCCT1 cells using si-circ_0021205-1 and si-circ_0021205-2 (Figure 2A) and up-regulated in KMBC cells using over-expression vectors (Figure 2B). si-circ_0021205-2 was selected for the subsequent experiments (Figure 2A). Results of CCK-8 assay showed that the down-regulation of circ_0021205 significantly inhibited CCA cell proliferation, whereas circ_0021205 up-regulation promoted the proliferation of CCA cells (Figures 2C,D). Colony formation assay indicated that circ_0021205 inhibition suppressed cell cloning capability of CCA cells, however, up-regulation of circ_0021205 exerted opposite effects (Figure 2E). We further confirmed the role of circ_0021205 in regulation of CCA cells proliferation using western blotting to detect the expression levels of proliferation makers, Ki-67 and PCNA (Figure 2F). In addition, transwell assay showed that the down-regulation of circ_0021205 inhibited the migration and invasion of CCA cells, whereas circ_0021205 up-regulation promoted CCA cell migration and invasion (Figure 2G). These results demonstrated that circ_0021205 promoted the proliferation, migration and invasion of CCA cells *in vitro*.

### Circ_0021205 Serves as a MiR-204-5p Sponge

**FIGURE 3:**
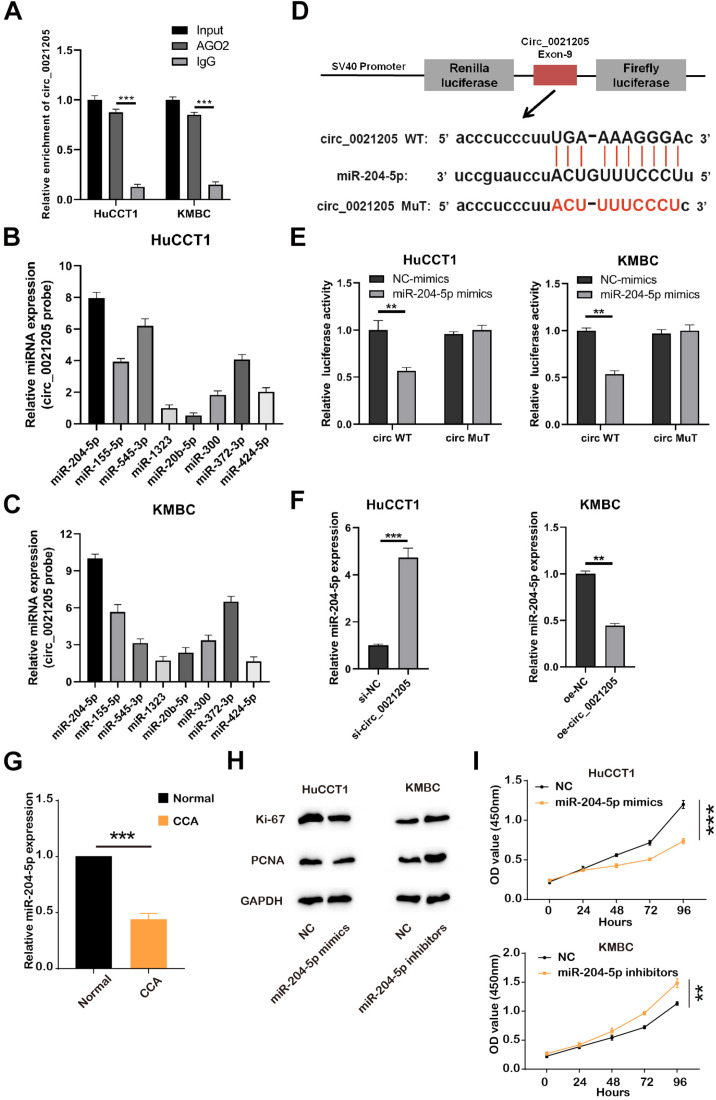
Circ_0021205 serves as a miR-204-5p sponge. **(A)** RIP experiments with AGO2 antibody were conducted, the expression of circ_0021205 was detected using qRT-PCR. **(B,C)** The expression of miRNAs bind to circ_0021205 probes in HuCCT1 and KMBC cells was detected by qRT-PCR. **(D)** Schematic illustration of circ_0021205-WT and circ_0021205-MuT luciferase reporter vectors and the binding sites between circ_0021205 and miR-204-5p. **(E)** The relative luciferase activities were detected in HuCCT1 and KMBC cells after co-transfection with circ_0021205-WT/MuT and miR-204-5p mimics/NC-mimics, respectively. **(F)** The expression of miR-204-5p was detected in HuCCT1 and KMBC cells transfected with si-circ_0021205 and OE-circ_0021205 plasmids, respectively. **(G)** Relative expression of miR-204-5p was detected in CCA and normal tissues. **(H)** The levels of proliferation makers, Ki-67 and PCNA, were investigated using western blotting. **(I)** The proliferation of transfected CCA cells was determined by CCK-8 assay. All experiments were repeated at least three time. **P < 0.01, ***P < 0.001.

To explore whether circ_0021205 sponging miRNAs, RIP experiments were performed. As shown in Figure 3A, the expression of circ_0021205 was enriched in AGO2 groups, indicating that circ_0021205 could bind to miRNAs through AGO2 protein. Then we predicted the potential target miRNAs through CircInteractome^1^, and detected their levels bind to specific circ_0021205 probe in HuCCT1 (Figure 3B) and KMBC (Figure 3C) cells. Results showed that the levels of miR-204-5p binding to circ_0021205 probe were higher in both HuCCT1 and KMBC cells. Next, miR-204-5p was selected for subsequent experiments. To further verify the interaction between circ_0021205 and miR-204-5p, luciferase reporter vectors were constructed (Figure 3D). And miR-204-5p mimics significantly decreased the luciferase activity in circ_0021205 WT groups, whereas brought no changes in circ_0021205 MuT groups (Figure 3E). The results demonstrated that circ_0021205 could directly bind to miR-204-5p. In addition, the expression of miR-204-5p was significantly increased in HuCCT1 cells transfected with si-circ_0021205, and circ_0021205 over-expression inhibited miR-204-5p expression in KMBC cells (Figure 3F). We then further examined the expression level of miR-204-5p using CCA tissues, and found that miR-204-5p level was remarkably decreased (Figure 3G). Through *in vitro* assays, we also confirmed that miR-204-5p mimics inhibited the proliferation of CCA cells, and miR-204-5p inhibitors enhanced the proliferation ability of CCA cells with detection of proliferation markers and CCK-8 assay (Figures 3H,I). These data illustrated that circ_0021205 serve as a miR-204-5p sponge.

### RAB22A Is a Direct Target of MiR-204-5p

**FIGURE 4:**
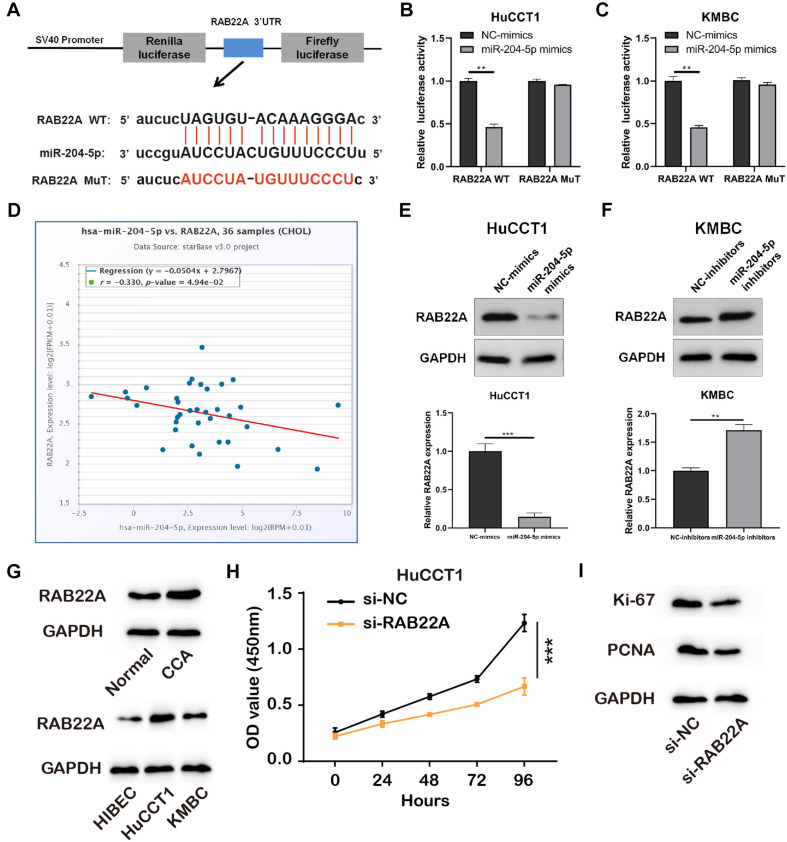
RAB22A is a direct target of miR-204-5p. **(A)** Schematic illustration of RAB22A-WT and RAB22A-MuT luciferase reporter vectors and the binding sites between RAB22A and miR-204-5p. **(B,C)** The relative luciferase activities were detected in HuCCT1 and KMBC cells after co-transfection with RAB22A-WT/MuT and miR-204-5p mimics/NC-mimics, respectively. **(D)** The correlation between miR-204-5p and RAB22A in CCA tissues. **(E,F)** The western blot was used to detect the expression of RAB22A in HuCCT1 and KMBC cells transfected with miR-204-5p mimics and miR-204-5p inhibitors, respectively. **(G)** The levels of RAB22A in CCA and normal tissues and CCA cell lines were investigated using western blotting. **(H)** The proliferation of transfected CCA cells was determined by CCK-8 assay. **(I)** The levels of proliferation makers, Ki-67 and PCNA, were investigated using western blotting. All experiments were repeated at least three time. **P < 0.01, ***P < 0.001.

We predicted target genes of miR-204-5p by Starbase 3.0, and RAB22A had been reported to involve in the progression of several cancers (Liao et al., 2020). The putative binding sites between miR-204-5p and RAB22A WT or MuT were shown in Figure 4A. Luciferase reporter vectors containing RAB22A WT/MuT sequences were co-transfected with miR-204-5p mimics/NC-mimics into HuCCT1 and KMBC cells. The results showed that miR-204-5p significantly reduced the luciferase activities in RAB22A WT groups, but no changes in RAB22A MuT groups (Figures 4B,C). In addition, the data from TGCA showed that the expression of miR-204-5p was negatively correlated with RAB22A levels in human CCA tissues (Figure 4D). We also confirmed that RAB22A level was increased in both CCA tissues abd CCA cell lines (Figure 4G). Furthermore, the expression of RAB22A was detected in HuCCT1 and KMBC cell transfected with miR-204-5p mimics and miR-204-5p inhibitors, respectively. The results showed that the up-regulation of miR-204-5p significantly inhibited RAB22A expression, whereas miR-204-5p down-regulation increased RAB22A expression (Figures 4E,F). Also, results collected using CCK-8 assay and proliferation makers measurement shown that si-RAB22A reduced the proliferation of CCA cells (Figures 4H,I). These data demonstrated that RAB22A is a target gene of miR-204-5p.

### Circ_0021205 Promotes CCA Cell Proliferation, Migration, and Invasion Through MiR-204-5p/RAB22A Axis

**FIGURE 5:**
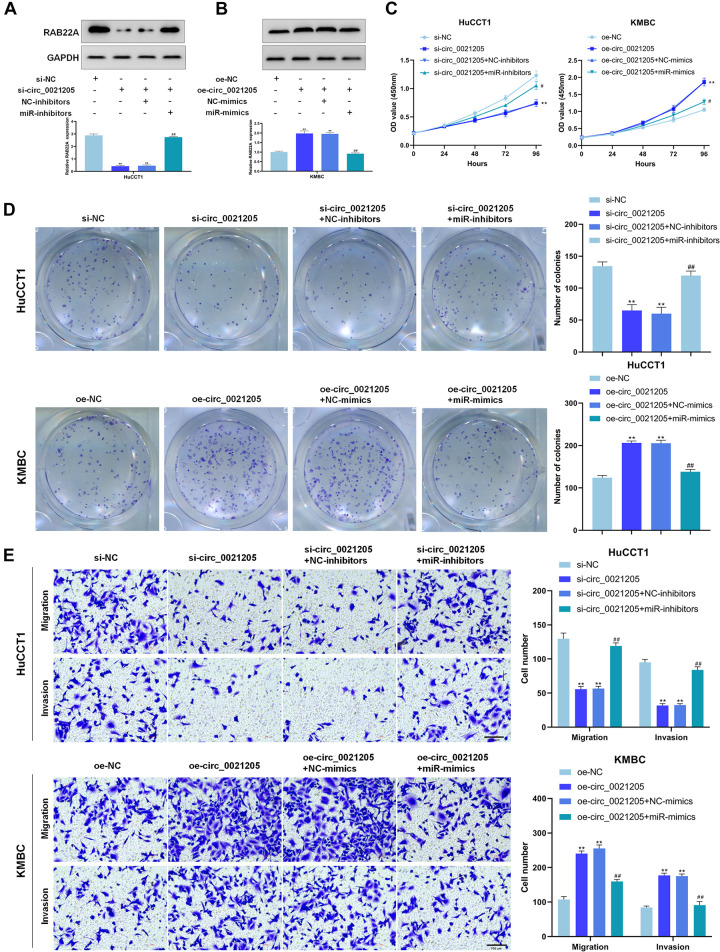
Circ_0021205 suppresses CCA cell proliferation, migration and invasion through miR-204-5p/RAB22A axis. **(A,B)** Western blot results showed that circ_0021205 regulated RAB22A expression through sponging miR-204-5p. **(C,D)** The proliferation ability of treated HuCCT1 and KMBC cells were detected by CCK-8 assay and colony formation assay. **(E)** Transwell assay was performed to detect the migration and invasion of treated HuCCT1 and KMBC cells. All experiments were repeated at least three time. **P < 0.01. # and ## mean the significant differences between oe-circ_0021205+miR-mimics and oe-circ_0021205+NC-mimics orsi-circ_0021205+miR-inhibitors and si-circ_0021205+NC-inhibitors.

To further explore whether circ_0021205 served as a tumor promoter through miR-204-5p/RAB22A axis, rescue experiments were performed using miR-204-5p mimics and inhibitors. Western blot assay showed that knockdown of circ_0021205 decreased RAB22A expression in HuCCT1 cells, while circ_0021205 up-regulation enhanced RAB22A expression in KMBC cells. Simultaneously, these effects caused by knocking down or over-expressing circ_0021205 were reversed by miR-204-5p inhibitors or mimics, respectively (Figures 5A,B). Moreover, CCK-8 assay (Figure 5C), colony formation assay (Figure 5D), and transwell assay (Figure 5E) were also performed. The results indicated that miR-204-5p inhibitors reversed the suppressing effects of circ_0021205 down-regulation on proliferation, migration, and invasion in HuCCT1 cells, whereas miR-204-5p mimics blocked the promoting effects induced by circ_0021205 over-expression in KMBC cells. Collectively, these results revealed that circ_0021205 served as a miR-204-5p sponge to regulate RAB22A expression, thus promoting the progression of CCA.

### Circ_0021205 Knockdown Inhibits the Tumorigenesis of CCA *in vivo*

**FIGURE 6:**
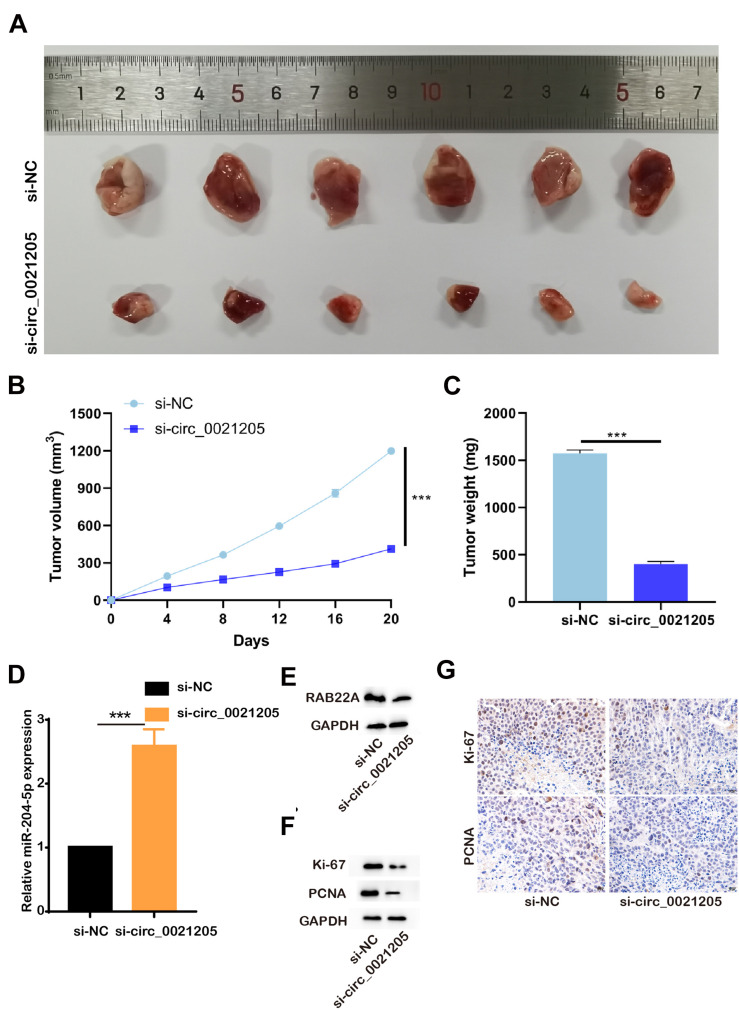
Circ_0021205 knockdown inhibits the tumorigenesis of CCA *in vivo*. **(A)** The presentative image of xenograft tumors was showed. **(B)** Tumor volumes were recorded every 4 days. **(C)** Tumor weights were recorded. **(D)** Relative miR-204-5p expression after down-regulation of circ_0021205 was measured using qPCR. **(E)** The levels of RAB22A in si-NC and so-circ_0021205 tumor tissues were investigated using western blotting. **(F)** The levels of proliferation makers, Ki-67 and PCNA, were investigated using western blotting. **(G)** The proliferation properties of si-NC and so-circ_0021205 tumor tissues were detected using immunohistochemistry staining. ***P < 0.001.

To further explore the role of circ_0021205 *in vivo*, the HuCCT1 cells transfected with si-NC or si-circ_0021205 were subcutaneously injected into the mice, respectively (*n* = 6). Tumor volumes were recorded every 4 days (Figure 6B) and the tumors were excised at 20 days after injection (Figure 6A). Then the tumor weights were recorded (Figure 6C). We then detect the expression level of miR-204-5p and RAB22A with qPCR and WB, and confirmed that si-circ_0021205 increased the expression level of miR-204-5p and decreased RAB22A level in tumor tissues (Figures 6D,E). Next, we investigated the proliferation of the cells in the tumors with western blotting and immunohistochemistry staining, and found the expression of Ki-67 and PCNA were significantly decreased after knocking down of circ_0021205 (Figures 6F,G). Six-month after the xenograft, we confirmed that si-circ_0021205 did not affect the mice survival. The results revealed that circ_0021205 knockdown could suppress the tumor growth of CCA *in vivo*.

## Discussion

In recent years, emerging evidence illustrates that many circRNAs function as tumor suppressors or promoters in several cancers, such as hepatocellular carcinoma, lung cancer, gastric cancer, and cholangiocarcinoma (Yang et al., 2019). So far, only a few CCA-related circRNAs have been recognized. In the present study, we first reveal that circ_0021205/miR-204-5p/RAB22A axis is involved in CCA progression.

In this study, we found that circ_0021205 was significantly up-regulated in CCA tissues and cells compare with the controls and associated with tumor size and TNM stage. Subsequently, the functional study demonstrated that circ_0021205 promoted the proliferation, metastasis, and invasion of CCA cells. Currently, accumulating studies reported that circRNAs could serve as miRNA sponges to regulate the expression of downstream genes. The “circRNA-miRNA-mRNA” axis has been reported to be involved in the development of multiple cancers. For example, circCCDC9 suppresses the progression of gastric cancer by regulating CAV1 via miR-6792-3p (Luo et al., 2020). Circ-ZKSCAN1 regulates FAM83A expression by sponging miR-330-5p to promote non-small lung cancer progression (Wang et al., 2019).

In our study, RIP experiment and liciferase report assay indicated that circ_0021205 could act as the sponge of miR-204-5p and RAB22A is the target gene of miR-204-5p. The rescue experiments demonstrated that circ_0021205 exerts carcinogenic effects in CCA by target RAB22A via miR-204-5p. RAB22A, a member of the RAB family of small GTPases, is known to be involved in several immune functions, and plays important roles in the endocytic recycling and the formation of T cell conjugates (Mayorga and Cebrian, 2019). Also, the expression of RAB22A determines the progression of multiple tumors, including liver cancer, ovarian cancer and malignant melanoma (Mayorga and Cebrian, 2019). In 2017, a study reported that Rab22a could enhance CD147 recycling and was required for the migration and invasion of lung cancer cells (Zhou et al., 2017). Given the above results, our results suggested the potential of circ_0021205 as a novel biomarker for the diagnosis of CCA. In CCA patients with elevated circ_0021205 level, circ_0021205, miR-204-5p and RAB22A could also be potential targets to be manipulated with the help of adeno-associated virus (AAV) to inhibit the progression of CCA. However, RAB22A plays important roles in multiple cancers containing liver cancer, ovarian cancer, malignant melanoma and CCA, and participates in multiple cell signaling pathways. So the manipulation of RAB22A may cause severe side effects in CCA patients. With the more specific manipulation of the upstream circ_0021205 and miR-204-5p, the side effects may be avoid. Nevertheless, there are some limitations in our study. Firstly, whether circ_0021205 can be stably detected in body fluids such as plasma still needs further study. Secondly, although the carcinogenic effects of circ_0021205 in CCA were revealed in this study, there also might be other critical circRNAs or mechanisms involved in the development of CCA. Therefore, the diagnostic potential of circ_0021205 in CCA is in need of further investigation.

In conclusion, this study demonstrates that circ_0021205 promotes CCA progression through miR-204-5p/RAB22A axis, which may provide a potential biomarker for CCA diagnosis.

## Data Availability Statement

The original contributions presented in the study are included in the article/supplementary material, further inquiries can be directed to the corresponding author/s.

## Ethics Statement

The studies involving human participants were reviewed and approved by the Ethics Committee of Lishui Hospital of Zhejiang University. The patients/participants provided their written informed consent to participate in this study. The animal study was reviewed and approved by the Ethics Committee of Lishui Hospital of Zhejiang University.

## Author Contributions

JT and WC designed this study. LZ, SF, DZ, CK, and YY performed these experiments. RQ, ZZ, and CL analyzed the data. XL and JJ wrote the manuscript. All authors contributed to the article and approved the submitted version.

## Conflict of Interest

The authors declare that the research was conducted in the absence of any commercial or financial relationships that could be construed as a potential conflict of interest.

## Footnotes

1 https://circinteractome.nia.nih.gov
